# Effectiveness of the Nutritional App “MyNutriCart” on Food Choices Related to Purchase and Dietary Behavior: A Pilot Randomized Controlled Trial

**DOI:** 10.3390/nu10121967

**Published:** 2018-12-12

**Authors:** Cristina Palacios, Michelle Torres, Desiree López, Maria A. Trak-Fellermeier, Catherine Coccia, Cynthia M. Pérez

**Affiliations:** 1 Department of Dietetics and Nutrition, Robert Stempel College of Public Health & Social Work, Florida International University, Miami, FL 33199, USA; ccoccia@fiu.edu; 2 Nutrition Program, Graduate School of Public Health, Medical Sciences Campus, University of Puerto Rico, San Juan 00935, Puerto Rico; michelle.torres13@upr.edu (M.T.); desire.lopeztravieso@upr.edu (D.L.); 3 Center for Clinical Research and Health Promotion, School of Dental Medicine, Medical Sciences Campus, University of Puerto Rico, San Juan 00935, Puerto Rico; maria.trak@upr.edu; 4 Department of Biostatistics and Epidemiology, Graduate School of Public Health, Medical Sciences Campus, University of Puerto Rico, San Juan 00935, Puerto Rico; cynthia.perez1@upr.edu

**Keywords:** nutritional application, smartphone, DGA, dietary behaviors, household food purchase behavior, obesity, overweight weight control

## Abstract

Objective: To pilot test the effectiveness of “MyNutriCart”, a smartphone application (app) that generates healthy grocery lists, on diet and weight. Methods: A pilot randomized trial was conducted to test the efficacy of using the “MyNutriCart” app compared to one face-to-face counseling session (Traditional group) in Hispanic overweight and obese adults. Household food purchasing behavior, three 24-h food recalls, Tucker’s semi-quantitative food frequency questionnaire (FFQ), and weight were assessed at baseline and after 8 weeks. Statistical analyses included *t* tests, a Poisson regression model, and analysis of covariance (ANCOVA) using STATA. Results: 24 participants in the Traditional group and 27 in the App group completed the study. Most participants were women (>88%), with a mean age of 35.3 years, more than a high school education (>80%), a family composition of at least three members, and a mean baseline body mass index (BMI) of 34.5 kg/m^2^. There were significant improvements in household purchasing of vegetables and whole grains, in individual intakes of refined grains, healthy proteins, whole-fat dairies, legumes, 100% fruit juices, and sweets and snacks; and in the individual frequency of intake of fruits and cold cuts/cured meats within the intervention group (*p* < 0.05). However, no significant differences were found between groups. No changes were detected in weight. Conclusions: “MyNutriCart” app use led to significant improvements in food-related behaviors compared to baseline, with no significant differences when compared to the Traditional group. Cost and resource savings of using the app compared to face-to-face counseling may make it a good option for interventionists.

## 1. Introduction

Diet-mediated chronic conditions affect half of the US adult population [1]. These could be prevented by following the science-based Dietary Guidelines for Americans (DGA) [2]. However, adherence to these guidelines is suboptimal [3]. In fact, several task forces have pinpointed the gap in translating the DGA recommendations into positive dietary changes [4,5,6], noting that the main barrier is the translation of the guidelines into practical, food-based recommendations and as such, new approaches are needed to implement these guidelines. In particular, innovative approaches should aim to improve grocery shopping, a critical moment when individuals need assistance for purchasing healthy foods [7]. With the huge food variety available in supermarkets, together with a large amount of nutritional information, and the limited time to read and understand nutrition labels, individuals may feel overwhelmed [8]. In fact, it has been reported that the main barrier to healthful shopping is a lack of self-efficacy in choosing healthy foods [9]. Therefore, interventions aimed at guiding individuals to choose healthy foods when grocery shopping may increase DGA adherence. This could be achieved by leveraging technology to help people make better choices at the point of purchase.

The use of tablets or smartphones for accessing the Internet is widespread, offering a unique platform for interventions. In 2016, 68% of all US adults owned a smartphone and 77% of them downloaded applications (apps) [10]. A myriad of nutrition and fitness apps have become extremely accessible via portable electronic devices with the capacity to calculate caloric requirements, track food intake and physical activity, and access healthy cooking information. In fact, studies have found better self-monitoring adherence and changes in dietary behaviors and/or weight control from using smartphone apps compared to traditional methods [11,12]. In addition to self-monitoring, a study found that “nudging” people to make healthy food purchases from local vendors resulted in improved awareness and consumption of healthy foods [11]. However, there are no available apps that translate the DGA into a healthy grocery list.

Therefore, in collaboration with technology experts, we developed the “MyNutriCart” app to help individuals make smart and healthy choices when purchasing foods at grocery stores [13]. This app automatically generates a healthy grocery list following DGA recommendations and accounts for the family’s nutritional needs, within a pre-specified budget [13]. The purpose of this study is to report on the pilot test of this app for improving household food purchase behavior and for improving individual dietary behaviors, compared to a traditional nutritional counseling face-to-face session in a convenience sample of overweight and obese Hispanic adults. As a secondary aim, we examined the potential effect of the intervention on weight control. We hypothesized that the use of the app would improve household food purchasing behavior when grocery shopping, which in turn would positively influence the individual frequency and intake of healthy foods and weight control compared to a traditional nutritional counseling session.

## 2. Materials and Methods

### 2.1. Study Design

We conducted a pilot randomized clinical trial to test the effectiveness of the “MyNutriCart” app on household food purchase behavior, individual dietary behaviors, and individual weight control. Recruited participants were randomly assigned to either the App group or the Traditional group for 8 weeks. Diet and weight were assessed at baseline and after 8 weeks of intervention. This time frame was chosen for this pilot study as this is the time frame used in similar studies using apps with significant changes in diet and/or weight. Recruitment was conducted between December 2015 and March 2016 and all study visits were conducted at the Medical Sciences Campus, University of Puerto Rico. The Institutional Review Board at this institution approved this study. Prior to the study, all recruited participants provided written consent.

### 2.2. Participants, Eligibility, and Recruitment

For this pilot trial, a convenience sample of participants was recruited between January–March of 2016 using flyers posted on the university intranet and around campus, shopping malls, clinics/medical offices, and by word of mouth. Overweight and obese adults aged 21–45 years were invited to participate in a study to test an app that helps individuals select healthier food, which could impact their dietary behaviors and weight control. Additional inclusion criteria were: being the main household shopper (i.e., responsible for >50% of the household grocery acquisition), shopping at a grocery store at least once weekly, owning a smartphone (iPhone or Android) with internet access, and willingness to be randomized into one of the two groups. We excluded those already using apps to monitor diet and/or physical activity or those enrolled in weight loss programs. Pregnant women, individuals with chronic health conditions (i.e., diabetes, kidney disease), or with reported food allergies were deemed ineligible.

### 2.3. Intervention Groups

Participants were equally randomized to either the App or Traditional groups using a simple computerized randomization scheme. Participants were assigned their allocation following a sequentially numbered container mechanism. Randomization was done by the statistician.

#### 2.3.1. “MyNutriCart” (App Group)

Participants allocated to the App group were guided by the research assistant in how to download and navigate the app. The MyNutriCart app was developed to guide individuals to make smart and healthy choices when purchasing foods at grocery stores, as recently published [13]. Briefly, the app provided a healthy grocery list based on the daily nutritional recommendations of the individuals that constitute the participant’s household. This list took into consideration a pre-defined budget, which was maximized by connecting to supermarkets’ discounts. It also integrated the following aspects:

- Estimation of energy requirements for each family member based on age, sex, and physical activity using the equations from the Dietary Reference Intakes [14]. The app automatically subtracted 500 kcals from the total calculated energy requirement for study participants only (not family members) to allow for a weight loss of about 1 pound/week [15];
- General food recommendations from the DGA [2], such as consumption of half of the grains as whole grains and low-fat dairy products, in addition to a variety of protein foods (beans, eggs, poultry, fish, and seashells);
- Number of servings per food group, based on the caloric level of each member, as recommended by the DGA [2]. Servings of each food group from each member were added to get a total of each food group per day;
- Intended number of days of the shopping event to multiply the servings per food group to get a total of foods to purchase;
- Participant’s pre-specified budget and weekly discounts offered by the largest local supermarkets (which was retrieved from an independent and free website service) to maximize the budget;
- Sample menus for each caloric level of the household based on local preferences, which were previously designed by a registered dietitian (RD).

The primary goal of the app use is the establishment of a healthy eating pattern; hence, energy dense items or foods containing added sugar (i.e., sweetened beverages, juices of any type, alcoholic beverages, sweets and desserts, and non-healthy snacks) were excluded from the grocery list. Only healthy versions for the following main food groups were included: fruits, vegetables, dairy products, cereals and grains, and protein foods. Participants were informed that this list would cover most of their energy requirements, but not all, as it excluded items not purchased weekly, such as fats and oils, and other items such as condiments, sauces, spices, coffee or tea, and bottled water. Participants were also instructed to use the app every time they went grocery shopping or at least once per week. To generate the list, the user had to open the app before each grocery event, select a budget amount, and a time frame for that grocery event (i.e., $100 for 7 days). The app then generates a grocery list for each supermarket included in the app based on their weekly specials. Therefore, each list generated was unique. Participants were free to choose from the supermarkets included in the app to do their grocery shopping, based on the convenience of its location, the total amount estimated to pay if all the foods were purchased, and the discounts offered that week. The app did not include notifications or reminders to use the app.

#### 2.3.2. Traditional Nutritional Counseling (Traditional Group)

This group received one face-to-face counseling session with an RD at the beginning of the study. The RD calculated the participant’s energy requirements using the Dietary Reference Intakes [14] and subtracted 500 kcals to allow for weight loss [15], similar to the App group. The RD provided the participant with the MyPlate Tip sheets [16], which are based on the DGA recommendations and contain the recommended food groups’ servings per caloric level. Also, participants received a sample menu, similar to the menu included in the app. There were no follow-up calls or additional sessions during the study.

During the study, all participants were instructed to maintain their usual physical activity level and to avoid partaking in other programs or sessions related to weight loss or promoting healthy dietary behaviors. Compliance with these study requirements was verified through a brief questionnaire at post-intervention.

### 2.4. Instruments and Measures

Trained research assistants conducted measures and interviews, as described below:

- Socio-demographics

A short questionnaire was completed at baseline with information about age, sex, educational level, and family composition (number, age, and sex of family members).

- Household food purchase frequency

This was evaluated from grocery receipts collected at baseline and post-intervention. This method has been previously validated to assess household food purchasing behavior [17]. In particular, the purchasing frequency of the following key DGA food groups was evaluated from each grocery receipt: fruits, vegetables, whole grains, 100% fruit juices, and sugar-sweetened beverages (SSB). These were the only food groups selected as they were easily identifiable by name from the grocery receipts. Participants were asked to provide all the grocery receipts available from their grocery events near the baseline visit and all of their grocery receipts during the study, either by uploading a picture of the grocery receipt in the app, sending scanned copies by email, or submitting hard copies. We reminded participants throughout the study to keep all their grocery receipts. Each time the food group was identified in the receipt, it was counted as a frequency of one. For example, if a receipt showed: grapes $1.05, oranges $2.33, and bananas $0.99, this was counted as 3 fruits. It was not possible to evaluate amount purchased as this information was not readily available from all grocery receipts. Results were averaged for each food group from the available receipts collected at baseline and at post-intervention.

- Dietary behaviors at the individual level

Participants were interviewed by trained research staff to complete the following questionnaires at baseline and at post-intervention:

- Food frequency questionnaire (FFQ). We used a short version of the Tucker’s semi-quantitative FFQ, which was validated in Puerto Rican adults [18]. The questionnaire was interview-administered, and respondents were asked to estimate the frequency of food consumption from 10 categories (daily, weekly, monthly), using the preceding eight weeks as the reference period. Summary questions for the frequency of consumption of the following food groups: fruits, vegetables, starchy vegetables, refined and whole grains, legumes, healthy proteins, red meats, cold cuts and cured meats, whole-fat and low-fat dairy products, 100% fruit juices, and SSB were conducted.
- Intake of foods using three 24-h dietary recalls. These were conducted during 2 non-consecutive weekdays and one weekend day using the Nutrition Data System for Research multi-pass method (5 steps) (Version 25, 2014) [19]. The baseline 24-h recalls were done before participants were informed about their group assignment; one was done in person at the baseline visit and the other 2 recalls were done by phone in the following 2–3 days. For the post-intervention recalls, we completed the first 2 by phone and the last one when they came to the post-intervention visit. For the first recall, we used a portion size booklet displaying standardized food servings as a visual aid for participants to estimate their usual portion sizes. A copy of this booklet was provided to each participant to take home to help in estimating portion sizes when we called them to complete the other recalls by phone. Intake (in servings) from the following food groups were averaged for the 3 days for both baseline and post-intervention recalls: fruits, vegetables, starchy vegetables, refined and whole grains, legumes, healthy proteins, red meats, cold cuts and cured meats, whole-fat and low-fat dairy products, 100% fruit juices, SSB, and snacks and sweets.

- Weight control at the individual level

Weight and height were assessed at baseline and at post-intervention (only weight). These measurements were taken with participants wearing light clothing, no shoes, hats, or any other objects that could cause interference. Weight was determined in kg using a calibrated scale (BF-350 TANITA, Arlington Heights, IL, USA) with a 0.1 kg accuracy. Height was measured in cm using a portable stadiometer, with a 0.1 cm accuracy (Charder HM200P Portable Stadiometer, Taichung, Taiwan). Measurements were taken in duplicates and averaged. Body mass index (BMI) was calculated as kg/m^2^.

### 2.5. Data Analysis

Descriptive statistics (frequency and percentage for categorical variables and mean (standard deviation) for continuous variables) were reported. Comparison between the App and Traditional groups at baseline and within group changes were performed using Student *t* tests. Analysis of covariance (ANCOVA) was used to assess differences between intervention groups for each outcome assessed, in which intake of foods (in servings) or frequency of food intake or weight/BMI were used as the dependent variables, group assignment as the fixed factor, and baseline value of the dependent variables as covariates. The effect sizes were calculated using the partial eta-squared, and the values 0.01, 0.06, and 0.14 were considered small, moderate, and large effects, respectively [20,21]. Due to the substantial proportion of zeroes in food purchase behavior data, a Poisson regression model was used to assess the effect of the intervention on the food selection after 8 weeks controlling for baseline values. All analyses were computed using Stata version 15 (StataCorp, College Station, TX, USA), and did not adjust for multiplicity nor missing value imputations.

## 3. Results

**Figure 1:**
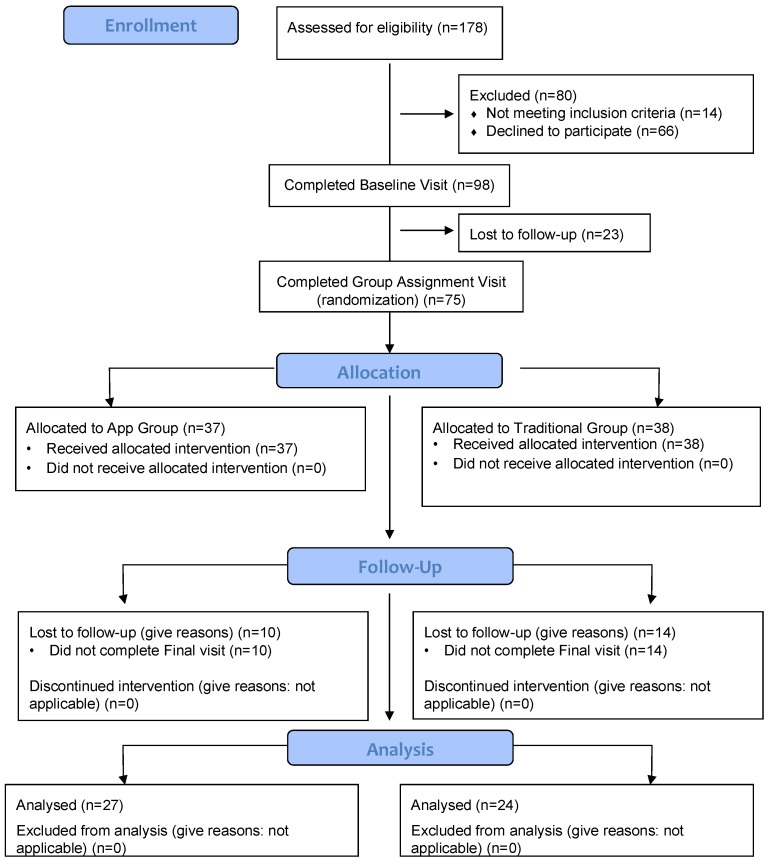
Participant flow chart.

A total of 37 participants were randomized to the App group and 38 to the Traditional group, as shown in Figure 1. Not all participants completed all aspects of the study. Within the Traditional group, 18 completed the FFQ, 17 completed the 24-h recalls, and 18 completed the grocery receipt collection. Within the App group, 25 participants completed the FFQ, 15 completed the 24-h recalls, and 13 completed the grocery receipt collection. A total of 17 (8 in the Traditional group and 9 in the App group) completed all aspects of data collection (three 24-h recalls, at least two receipts, the FFQ, and weight measurements, both at baseline and post-intervention). Table 1 summarizes the characteristics of those who completed at least one aspect of the study. No differences were observed in baseline characteristics between intervention groups. Most participants were women (>88%), mean age was 35.3 years, most had more than high school education (>80%), a family composition of at least three members, and a mean baseline BMI of 34.5 kg/m^2^. Also, no differences were observed in any of the baseline characteristics between those who completed or fail to complete the study (data not shown).

Results for household food purchase frequency are shown in Table 2. Compliance with grocery receipts submission was low, therefore, the analysis included participants that had submitted at least two grocery receipts at baseline and post-intervention. No differences were observed at baseline between intervention groups. Within groups, we observed a significant increase in the frequency of purchase of vegetables and whole grains in the App group (*p* < 0.05) from baseline to post-intervention. We also analyzed the change of household food purchase frequency during the 8 weeks of the study using Poisson regression, adjusting for food purchase behavior at baseline. The coefficient associated with the intervention (App vs. Traditional) is the expected difference in log count between the App group and the Traditional group. Compared to the Traditional group, the estimated Poisson regression coefficient was 0.27 for fruits (standard error [SE] = 0.26; *p* = 0.29), 0.05 for vegetables (SE = 0.19; *p* = 0.79), 0.46 for whole grains (SE = 0.46; *p* = 0.41), 1.36 for 100% fruit juices (SE = 0.78; *p* = 0.08), and 0.51 for SSB (SE = 0.51; *p* = 0.09).

Individual food intake, as assessed from three 24-h recalls, is shown in Table 3. At baseline, the App group consumed significantly fewer servings of whole-fat dairy foods compared to the Traditional group. Within groups, we observed a decrease in the intake of refined grains, healthy proteins, and whole-fat dairy products in the Traditional group (*p* < 0.05) and a significant decrease in the intake of refined grains, legumes, 100% fruit juices, and sweets and snacks in the App group (*p* < 0.05) from baseline to post-intervention. However, when analyzing the change in food intake using ANCOVA to adjust for baseline data, as shown in Table 4, only a trend for a significant decrease in the intake of legumes in the App group compared to the Traditional group (*p* = 0.06) was observed. We also assessed individual food frequency from the FFQ, as shown in Supplementary Table S1, and found that at baseline, the App group consumed low-fat dairy foods with less frequency compared to the Traditional group (*p* < 0.05). Within groups, we observed a decrease in the frequency of intake of cold cuts and cured meats in the Traditional group (*p* = 0.05) and a significant increase in the frequency of intake of fruits in the App group (*p* < 0.05) from baseline to post-intervention. However, when analyzing changes in frequency of food intake using ANCOVA to adjust for baseline data, only a trend for an increase in the frequency of consumption of whole grains (*p* = 0.08) and a significant increase in the frequency of consumption of cold cuts and cured meats in the App group compared to the Traditional group (*p* = 0.01) was observed.

For weight and BMI, there were no differences between or within groups, as shown in Supplementary Table S2. No harm or unintended effects were observed in either of the allocation groups.

Results on the evaluation of the app have been previously published [13]. Briefly, the exit interview at post-intervention showed that most (>50%) considered the app to be feasible, acceptable, usable at least once in the last month and they were satisfied; the short survey completed by participants at the end of their grocery shopping (*n* = 23) showed that 73.1% used the app every time they went grocery shopping and that 26.1% purchased ≥70% of the recommended products in the list.

## 4. Discussion

This is the first study to test an app that generates a shopping list based on energy requirements, following the DGA and accounting for budget and supermarkets’ discounts. Those using “MyNutriCart” purchased vegetables and whole grains significantly more frequently at the household level, while at the individual level they significantly consumed more servings of refined grains, legumes, 100% fruit juices, and sweets and snacks and significantly consumed fruits more frequently at post-intervention compared to baseline. However, the Traditional group also had some improvements, so when analyzing changes in these behaviors during the study between groups using Poisson regression or ANCOVA, the App group only had a significantly greater frequency of consumption of whole grains and cold cuts and cured meats with a lower intake of legumes compared to the Traditional group. No effects on weight control were detected.

As hypothesized, “MyNutriCart” improved some aspects of household food purchasing behavior (i.e., higher vegetables and whole grains purchase), which translated into a lower intake of refined grains at the individual level. However, it is interesting to note that purchasing vegetables more frequently at the household level did not translate into a greater intake of vegetables at the individual level, although intake did improve somewhat compared to baseline. Since this is a measure of the household food purchase frequency, it may explain why it did not specifically translate to greater vegetable consumption at the individual level. However, compared to the Traditional group, none of the changes regarding household food purchases were significant. This was not expected as the app considered the household budget, the supermarkets’ weekly discounts, and only included in the shopping list only those fruits and vegetables offered at a reduced price, to maximize the budget as the price of fresh produce varies considerably depending on the season. Therefore, the app showed participants that healthy foods could be purchased even within a tight budget. Certain food purchasing behaviors are easier to be influenced, such as purchasing whole grains as they are readily available in all supermarkets and most refined grains (i.e., white rice, white bread, white tortillas), have a healthier whole grain option (i.e., brown rice, whole multigrain bread, whole-wheat tortillas). In fact, other studies aiming to improve diet quality have found improvements in whole grains [22], therefore, switching from refined to whole grains seems to be easier than introducing new foods, such as fruits or vegetables. In particular, consumption of fruits and vegetables was low in both groups and improvements were observed in the App group including a greater frequency of consumption of fruits (*p* = 0.02) and a trend in a greater number of servings of vegetable consumed (*p* = 0.06) at post-intervention compared to baseline, which is consistent with other studies conducted in similar groups [23,24,25,26]. Other trials targeting fruits and vegetables among populations with traditionally low intakes have also found significant improvements [27,28]. These products are often perceived as expensive [29,30]; which is the main reason our app only included in the shopping list the fresh produce that was on sale that week. However, more intensive interventions may be needed to increase household purchasing of fruits and vegetables and to translate this into a higher individual intake of these foods.

Currently, there are a limited number of studies investigating the purchase of healthy foods in grocery stores and improvement in dietary behaviors using a smartphone app, although there are a few trials that are currently ongoing. A study among 208 adults in Canada testing the “SmartAPPetite” app for 8-10 weeks found a significant decrease in the intake of soft drinks, sugary and fast foods and an increase in homemade meals and fruits, particularly among those using the app more frequently [11]. Also, 46% of participants believed that the messaging changed their food purchasing habits [11]. A study testing an app to improve vegetables among 135 overweight adults for 8 weeks found a significantly greater vegetable intake among the intervention group compared to the control group [31]. Another trial testing the effect of a “SaltSwitch” app among 66 adults with cardiovascular disease for four weeks found a significant reduction in salt purchase, which resulted in a reduction of 0.7 g of salt/day per person, compared to the usual care group [32]. Most of these trials showed that compliance was reduced over time and that those that were more compliant with the intervention (i.e., greater use of the app) had greater outcome effects. However, results from these trials provide evidence on the effects of such apps in improving food selection and purchase, although more studies are needed to understand how individuals use the apps.

Although there are only a few trials testing apps to improve household food purchasing behavior and dietary behaviors, they have the potential to support/reinforce adherence to the DGA. However, this technology may be insufficient for helping individuals make the necessary behavioral changes. As found in our study, only one intervention session without follow-ups or app notifications to remind participants to use the app, led to only a few significant improvements in dietary behaviors. More intensive follow-ups with app notifications may be needed to facilitate behavioral change. Some participants may need more counseling than others, therefore, sessions should be personalized depending on the level of behavioral change needed by each participant. Also, follow-up sessions may be necessary to keep participants motivated in using the app, as we previously reported that only 26% purchased more than 70% of the items recommended on the grocery list at each shopping event [13]. Others have reported that greater app interactions led to greater dietary changes [11]. The low app use in the present study could also explain the lack of greater changes in the study and also on the lack of effects on weight; as evidenced by others [33]. Studies testing apps for weight control have found significant effects [34,35], particularly those using more intensive approaches [12,35] and even among short-term studies [34,35]. Therefore, interventions using smartphone apps may require several sessions/calls/follow-ups during the study to maintain motivation towards using the app.

The present study helped identify the potential of the “MyNutriCart” app to improve household food purchase and individual dietary behaviors using a randomized clinical trial design; with trained research assistants and using validated tools. It also helped identify limitations that should be considered in future investigations, such as its short duration and small sample size. Another limitation was the lack of follow-up messages or app notifications to remind participants to use the app. We did not assess their prior experience with healthy eating, which could have affected our results. The information from grocery receipts was limited to frequency, as amount purchased was not readily available from all grocery receipts. We did not assess if the frequency of grocery shopping changed with the study, which could have affected the selection of foods. We also did not assess how many members of the household each shopping event was intended for; however, the app did take into account the number of members in the household when coming up with the list. In addition, due to low compliance with grocery receipts submission, the analysis was based on at least two receipts at each time, which may not be representative of usual household purchase. We also did not ask if other household members did complementary grocery shopping during the study, which should be accounted for in future studies. “MyNutriCart” was tested among Hispanics and integrated elements of Hispanic diets, but its conception is based on the DGA, hence its applicability does not exclude other ethnic groups. Future studies should also integrate all family members, as the app provides a healthy grocery list for the entire family and we learned at the end of the study that some family members disliked some of the recommended foods, as previously reported [13].

## 5. Conclusions

In conclusion, the use of the “MyNutriCart” app led to small improvements in household food purchase and individual food intake over the 8-week period compared to the initial assessment but there were basically no significant improvements compared to the Traditional group. Therefore, these results may suggest that the “MyNutriCart” app is as good as the traditional method for improving these behaviors. Using such tools could reduce costs and resources for improving household food purchase and dietary quality. Also, these tools may help reach out to other target groups, that may not reach out to health professionals for improving their diet. However, neither of the interventions led to changes in weight control. More intense interventions with greater follow-up visits, app notifications, calls or messages are needed to achieve greater changes in food-related behaviors and weight outcomes. In the future, larger and longer trials with more intensive follow-ups may be needed to detect changes in the desired outcomes.

## Figures and Tables

**Table 1:** Baseline characteristics of study participants by intervention groups (*n* = 51).

Variable	Traditional group (*n* = 24)	App group (*n* = 27)	*p* Value *
	Mean (SD) or %		
Age, years	36.8 (5.86)	33.8 (7.30)	0.12
Female sex, %	91.7	88.9	0.56
More than high school education, %	83.3	81.5	0.58
Number of family members in household	3.17 (1.24)	3.11 (1.34)	0.88
Weight (kg)	83.3 (14.9)	93.3 (20.4)	0.09
Height (m)	1.58 (0.06)	1.62 (0.08)	0.12
BMI, kg/m^2^	33.3 (5.81)	35.6 (7.50)	0.29
Overweight, %	31.6	30.0	0.92
Obese, %	68.4	70.0	

SD: standard deviation; BMI: body mass index. * *t* test. Level of significance was *p* < 0.05.

**Table 2:** Household food purchase behavior at baseline and post-intervention by groups (*n* = 31) ^†^.

Variable	Baseline				Post-Intervention				Difference between Groups at Baseline	Difference between Baseline and Post-Intervention	
	Traditional Group (*n* = 18)		App Group (*n* = 13)		Traditional Group (*n* = 18)		App Group (*n* = 13)			Traditional Group	App Group
	Mean	SD	Mean	SD	Mean	SD	Mean	SD	*p* Value *	*p* Value *	
Fruits	1.03	1.02	1.27	1.05	1.78	1.63	2.34	2.31	0.53	0.05	0.08
Vegetables	1.75	1.76	1.31	1.03	3.42	4.07	3.71	3.68	0.43	0.07	0.02
Whole grains	0.31	0.55	0.23	0.39	0.61	0.49	0.98	1.37	0.68	0.06	0.04
100% fruit juices	0.14	0.33	0.08	0.19	0.13	0.28	0.49	0.94	0.55	0.45	0.07
SSB ^††^	1.14	1.19	0.65	0.85	1.10	1.25	2.13	3.46	0.22	0.46	0.10

^†^ Data estimated from two shopping receipts at baseline and at least two shopping receipts during the last 2 weeks of the intervention; ^††^ SSB: sugar sweetened beverages; SD: standard deviation. * *t* test. Level of significance was *p* < 0.05.

**Table 3:** Individual intake of food (servings/day) at baseline and post-intervention by groups (*n* = 32) ^†^.

Variable	Baseline				Post-Intervention				Difference between Groups at Baseline	Difference between Baseline and Post-Intervention	
	Traditional Group (*n* = 18)		App Group (*n* = 13)		Traditional Group (*n* = 18)		App Group (*n* = 13)			Traditional Group	App Group
	Mean	SD	Mean	SD	Mean	SD	Mean	SD	*p* Value *		
Fruits	0.87	0.96	1.13	1.16	1.09	0.99	1.37	1.06	0.49	0.19	0.18
Vegetables	0.65	0.69	0.76	0.69	0.69	0.69	1.45	1.55	0.66	0.43	0.06
Starchy vegetables	1.18	0.94	1.10	0.65	1.10	0.93	1.60	1.28	0.78	0.39	0.10
Refined grains	3.59	2.06	3.36	1.38	2.64	1.65	2.18	1.51	0.71	0.02	0.01
Whole grains	1.12	0.73	1.32	1.03	1.41	0.79	1.78	1.17	0.52	0.06	0.09
Legumes	0.13	0.16	0.23	0.24	0.18	0.18	0.07	0.12	0.14	0.16	0.02
Healthy proteins	1.26	1.20	0.82	0.84	0.77	1.04	0.42	0.59	0.24	0.05	0.10
Red meats	3.90	1.94	4.25	1.98	3.49	1.92	4.22	1.76	0.62	0.23	0.48
Cold cuts & cured meats	0.40	0.47	0.38	0.51	0.50	0.47	0.40	0.44	0.91	0.22	0.46
Whole-fat dairies	1.01	0.58	0.42	0.31	0.63	0.35	0.38	0.28	0.00	0.00	0.34
Low-fat dairies	0.36	0.25	0.75	0.75	0.43	0.60	0.75	0.87	0.06	0.33	0.49
100% fruit juices	0.23	0.41	0.35	0.47	0.09	0.16	0.11	0.28	0.47	0.11	0.01
SSB ^††^	0.45	1.29	0.07	0.15	0.24	0.46	0.08	0.19	0.27	0.28	0.41
Sweets and snacks	1.32	1.57	1.36	1.05	0.98	1.16	0.58	0.74	0.94	0.21	0.03

^†^ Data collected from three 24-hrs food recalls at baseline and three 24-hrs foods recalls at the end of the study; includes nuts, fish, and poultry; ^††^ SSB: sugar sweetened beverages; SD: standard deviation. * *t* test. Level of significance was *p* < 0.05.

**Table 4:** Analysis of covariance for individual food intake (servings/day) at 8 weeks (Traditional group *n* = 17; App group *n* = 15).

Variable	Adjusted Mean Difference	95% CI	*p* Value *	Partial Eta-Squared
Fruits	0.13	−0.50, 0.77	0.67	0.006
Vegetables	0.74	−0.12, 1.60	0.09	0.10
Starchy vegetables	0.52	−0.29, 1.32	0.20	0.06
Refined grains	−0.35	−1.34, 0.64	0.47	0.02
Whole grains	0.27	−0.38, 0.93	0.40	0.02
Legumes	−0.11	−0.23, 0.004	0.06	0.12
Healthy proteins	−0.25	−0.88, 0.37	0.41	0.02
Red meats	0.68	−0.67, 2.04	0.31	0.04
Cold cuts and cured meats	−0.10	−0.42, 0.23	0.55	0.01
Regular dairies	−0.09	−0.34, 0.17	0.49	0.02
Low-fat dairies	0.35	−0.23, 0.93	0.22	0.05
100% fruit juices	−0.005	−0.16, 0.15	0.94	0.0002
SSB ^†^	−0.17	−0.44, 0.10	0.21	0.05
Snacks and sweets	−0.40	−1.13, 0.32	0.26	0.04

Includes nuts, fish, and poultry; ^†^ SSB: sugar sweetened beverages. * Analysis of covariance (ANCOVA) was used to assess differences between intervention groups, with food intake as the dependent variable, group assignment as the fixed factor, adjusting for food intake at baseline. Level of significance was *p* < 0.05.

## Supplementary Materials

The following are available online at http://www.mdpi.com/2072-6643/10/12/1967/s1, Table S1: Frequency of food consumption at baseline and post intervention by groups (*n* = 43), Table S2: Weight and BMI of study participants at baseline and post intervention by groups (*n* = 37).
