# Supplementary material for: Effectiveness of the Nutritional App “MyNutriCart” on Food Choices Related to Purchase and Dietary Behavior: A Pilot Randomized Controlled Trial
Source: Nutrients. 2018 Dec 12;10(12):1967. doi: 10.3390/nu10121967 (PMC6315974; doi:10.3390/nu10121967)
Supplement: Supplementary file 1 [file nutrients-10-01967-s001.pdf]

Supplementary Materials

Table S1. Frequency of food consumption at baseline and post intervention by groups (*n* = 43) †.

Variable	Baseline				Post Intervention				Difference between Groups at Baseline	Difference between Baseline and Post Intervention		Adjusted Mean Difference	95% CI	*p* value **	Eta-squared
	Traditional group (*n* = 18)		App group (*n* = 25)		Traditional group (*n* = 18)		App group (*n* = 25)			Traditional Group	App Group				
	Mean	SD	Mean	SD	Mean	SD	Mean	SD	*p* value *						
Fruits	0.49	0.50	0.67	0.58	0.69	0.57	0.94	0.72	0.29	0.06	0.02	0.14	-0.22, -0.49	0.44	0.02
Vegetables	0.89	0.68	0.76	0.65	0.78	0.63	0.92	0.61	0.55	0.44	0.30	0.18	-0.18, 0.54	0.32	0.03
Starchy vegetables	0.43	0.47	0.58	0.56	0.34	0.26	0.37	0.33	0.34	0.75	0.59	0.01	-0.18, 0.20	0.92	0.0003
Refined grains	1.50	0.84	1.77	2.87	1.02	0.86	1.05	0.77	0.70	0.86	0.86	0.03	-0.48, 0.54	0.92	0.0003
Whole grains	0.59	0.56	0.64	0.51	0.60	0.46	0.91	0.60	0.77	0.20	0.18	0.28	-0.03, 0.59	0.08	0.08
Legumes	0.55	0.48	0.42	0.30	0.46	0.46	0.34	0.32	0.29	0.27	0.16	-0.08	-0.31, 0.15	0.50	0.01
Healthy proteins ¶	1.50	1.18	1.19	0.65	1.17	0.58	1.05	0.42	0.26	0.51	0.83	-0.03	-0.31, 0.25	0.82	0.001
Red meats	0.46	0.31	0.34	0.31	0.29	0.48	0.27	0.29	0.23	0.47	0.37	0.02	-0.21, 0.25	0.85	0.001
Cold cuts & cured meats	0.43	0.43	0.33	0.29	0.17	0.26	0.37	0.30	0.36	0.05	0.10	0.23	0.06, 0.40	0.01	0.15
Whole-fat dairies	0.89	0.61	0.60	0.55	0.48	0.53	0.52	0.44	0.11	0.73	0.73	0.09	-0.22, 0.40	0.56	0.01
Low-fat dairies	0.39	0.44	0.78	0.68	0.45	0.39	0.67	0.56	0.04	0.08	0.15	0.16	-0.17, 0.48	0.33	0.02
100% fruit juices	0.44	0.55	0.27	0.28	0.21	0.30	0.21	0.30	0.20	1.00	0.83	0.03	-0.16, 0.21	0.77	0.002
SSB ††	0.87	0.68	0.68	0.66	0.59	0.59	0.63	0.62	0.36	0.90	0.77	0.12	-0.23, 0.47	0.49	0.01

† Data collected from FFQ at baseline and at the end of the intervention; ¶ includes nuts, fish, and poultry; †† SSB: sugar sweetened beverages; SD: standard deviation. * *t* test. ** ANCOVA was used to assess differences between intervention groups, with frequency of each food consumption as the dependent variable, group assignment as the fixed factor, adjusting for frequency of food consumption at baseline. Level of significance was $p < 0.05$.

Table S2. Weight and BMI of study participants at baseline and post intervention by groups (*n* = 37) †.

Variable	Baseline				Post Intervention				Difference between Groups at Baseline	Difference between Baseline and Post Intervention	
	Traditional group (*n* = 19)		App group (*n* = 18)		Traditional group (*n* = 19)		App group (*n* = 18)			Traditional group	App group
	Mean	SD	Mean	SD	Mean	SD	Mean	SD	*p* value *		
Weight (kg)	83.3	14.9	93.3	20.4	82.8	15.2	92.9	21.5	0.09	0.12	0.27
BMI (kg/m2)	33.3	5.8	35.6	7.5	33.1	6.0	35.4	7.8	0.29	0.13	0.25

SD: standard deviation. * *t* test. * ANCOVA was used to assess differences between intervention groups, with weight or BMI at the end of study as the dependent variable, group assignment as the fixed factor, adjusting for weight and BMI at baseline. Adjusted mean difference for weight was -0.29 (95%CI -1.86, 1.28; p value 0.71; Eta-squared 0.004). Adjusted mean difference for BMI was 0.11 (95%CI -0.42, 0.64; p value 0.68; Eta-squared 0.005). Level of significance was $p < 0.05$.
